# Long-term population-based risks of second malignant neoplasms after childhood cancer in Britain

**DOI:** 10.1038/sj.bjc.6602226

**Published:** 2004-11-09

**Authors:** H C Jenkinson, M M Hawkins, C A Stiller, D L Winter, H B Marsden, M C G Stevens

**Affiliations:** 1 Department of Paediatric Oncology, Birmingham Children's Hospital NHS Trust, Birmingham B4 6NH, UK; 2 Department of Public Health and Epidemiology, Centre for Childhood Cancer Survivor Studies, University of Birmingham, Edgbaston, Birmingham B15 2TT, UK; 3 Childhood Cancer Research Group, Department of Paediatrics, University of Oxford, Oxford OX2 6HJ, UK; 4 Department of Paediatric Pathology, Royal Manchester Children's Hospital, Pendlebury, Manchester M27 4HA, UK; 5 Department of Paediatric Oncology, Bristol Royal Hospital for Children, Bristol BS2 8BJ, UK

**Keywords:** second malignant neoplasm, childhood cancer

## Abstract

In a population-based, retrospective cohort study of 16 541 3-year survivors of childhood cancer treated in Britain up to the end of 1987, 278 second malignant neoplasms (SMNs) were identified against 39.4 expected giving a standardised incidence ratio (SIR) of 6.2. The overall cumulative risk of an SMN by 25 years from 3-year survival from childhood cancer was 4.2%. Analysis of the cohort of nonretinoblastoma childhood cancers combined revealed a significant decline in SIR of SMN with increasing duration of follow-up. There was a greater risk of developing a SMN, particularly secondary acute myeloid leukaemia, in those diagnosed with childhood cancer from 1980 onwards. However, on multivariate modeling, this was not an independent risk factor. There was significant heterogeneity (*P*<0.001) in SIR of SMN across different treatment groups, the greatest risk observed in the group exposed to both radiotherapy and chemotherapy. The risks of SMN observed were comparable with those in other population-based studies. While the decline in SIR with duration of follow-up and the small excess numbers of cancers observed over later decades after diagnosis are reassuring, the high excess risk, particularly of leukaemia, associated with recent more intense therapy is of concern.

Survival after childhood cancer has greatly improved over the last three decades and most recent figures indicate that over 70% of children with cancer are likely to survive at least 5 years (National Registry of Childhood Tumours, unpublished). This growing population of survivors, estimated at 1 in every 1000 young adults (Hawkins and Stevens, 1996), is at risk of certain adverse late effects of both the cancer and its treatment including second malignant neoplasms (SMN). This long recognised increased risk (Meadows *et al*, 1985) represents perhaps the greatest challenge to long-term survival (Robison and Mertens, 1993). Of multifactorial aetiology, risk has been associated with the primary malignancy, exposure to chemotherapy and radiotherapy and genetic predisposition (Kony *et al*, 1997). A major difficulty in the analysis of SMNs is the assembling of large numbers of survivors who have been followed up over sufficiently long periods of time with a wide spectrum of treatments.

We have investigated the risks of SMNs after childhood cancer using a large UK population-based cohort of patients with substantially longer average follow-up than that in previous comparable studies.

## MATERIALS AND METHODS

### Case ascertainment

The cohort was selected from the National Register of Childhood Tumours (NRCT), a population-based national register covering the whole of Great Britain, which is maintained by the Childhood Cancer Research Group (CCRG) at the University of Oxford. The registry is notified of all cancers occurring in individuals aged less than 15 years through the national cancer registration system in Britain, which was established in 1962. In addition, a complementary series of 3-year survivors of childhood cancer diagnosed prior to this date was constructed from case lists from hospitals and tumour registries that were known to be complete.

SMNs were ascertained by several methods in order to obtain the most accurate estimation of the risk. Firstly, members of the cohort were ‘flagged’ at the National Health Service Central Registers (NHSCR), which provides automatic notification of the registration of death or cancer in these individuals (Hawkins and Swerdlow, 1992). At the time of finalising the cohort, virtually all cancer registrations up to 1990 had been processed at NHSCR and notified to researchers. Secondly, a series of postal questionnaires about SMNs were sent to the family doctors of childhood cancer survivors over the period from 1982 to 1990; these provided an independent source of ascertainment for 82% of the person-years of follow-up. Finally, the CCRG receive all death certificates that mention neoplasia in patients aged less than 20 years in Britain. These are routinely checked, through family doctor and hospital notes, to identify cases of multiple primary tumours. Cases were selected for the cohort if they had been diagnosed with a malignant neoplasm before 1st January 1988, aged less than 15 years at diagnosis and subsequently survived at least 3 years. SMNs were included if they were diagnosed before the end point of the study on 31st December 1990.

### Pathological criteria

For each individual in the cohort, the first primary tumour (FPT) was classified according to the International Classification of Diseases for Oncology (ICDO) (World Health Organization, 1976) and subsequently categorised by the diagnostic group (Birch and Marsden, 1987). SMNs were classified according to ICDO (World Health Organization, 1976). In addition, all SMNs were classified according to the International Classification of Diseases Ninth Revision (World Health Organization, 1979), as national incidence figures for this tumour classification are available for the general population, stratified by sex, calendar year and 5-year age groups, allowing statistical comparison. In all cases of a suspected SMN, pathological material from the first and second cancers was reviewed, if available, by a central pathologist (HBM).

### Statistical methods

Statistical tests and confidence intervals were based on the assumption that the observed numbers of cancers followed a Poisson distribution with a mean equal to the expected number of cases. Person-years at risk were accumulated from entry into the study (at 3-year survivorship) to exit from risk (first occurrence of SMN, died, emigrated or reached 31/12/90). The expected numbers of cancers were estimated by multiplying person-years at risk within specific categories defined by age (5-year groups), sex and single calendar year (from 1971) by the corresponding cancer incidence rate in the general population (Breslow and Day, 1987; Office of National Statistics, 1999). Standardised incidence ratios (SIR), the ratio of observed to expected number of cancers, and the additive excess risk (AER) were calculated. The AER is based on the difference between the observed and expected number of SMNs and is a measure of the excess number of cancers per 1000 survivors per year (Breslow and Day, 1987). Nonmelanomatous skin cancer (ICD 8 and 9: 1730–1739) was excluded from analysis, as being under-reported to cancer registries, their inclusion would create bias. Statistical tests of heterogeneity and trend in SIR were undertaken using standard methods described by Breslow and Day (1987). The cumulative risk was calculated using standard lifetable methods (Collett, 2003).

Multivariate Poisson regression models were used which simultaneously accounted for the effect of treatment (neither radiotherapy (RT) nor chemotherapy (CT)/RT alone/CT alone/both RT and CT), era of first cancer diagnosis (pre-1970/1970–1979/1980–1987), age at first cancer diagnosis (0–4/5–9/10–14 years) and length of follow-up on SMN risk (3–9/10–19/20–29/30 years or more) (Breslow and Day, 1987). Results are reported for all SMNs combined (excluding skin), as the models failed to converge for every specific type of SMN reported in Tables 4 and 5.

## RESULTS

In all, 16 541 cases of childhood cancer fulfilled the criteria for inclusion in the overall cohort showing a male to female ratio of 1.2. The FPT were diagnosed between 1926 and 1987 with a mean age at diagnosis of 6 years and 8 months (median; 5 years and 10 months). In total, 165 879 person-years of follow-up were accumulated during the study period, a mean follow-up per case of 10 years. Of the 16 541 3-year survivors in the cohort, 12 932 (78%) were followed up to the end point of the study. A total of 3331 (20%) survivors were censored before the end point (210 emigrated and 3121 died), and 278 (2%) developed an SMN.

The relative frequencies of first primary childhood cancers among individuals who developed an SMN were: central nervous system tumours (24.5%), retinoblastoma (15.8%), Hodgkin's disease (12.9%), Wilm's tumour (8.3%) and acute lymphoblastic leukaemia (7.2%). Within the entire cohort of 3-year survivors, 278 SMNs (with ICD9 codes 1400-2089) were observed, of which 33 were nonmelanomatous skin cancers, leaving 245 cases for analysis, compared to 39.4 expected, giving a SIR of 6.2 (95% CI: 5.5, 7.1). Table 1

**Table 1:** Risk of developing an SMN and selected sites in 3-year survivors of childhood cancer

	**Whole cohort (*n*=16 541)**		
**SMN sites and ICD9 codes**	**O**	**SIR**	**95% CI for SIR**
All (exc skin) 1400–2089 (exc 1730–1739)	245	6.2	5.5, 7.1
Digestive 1400–1599	32	9.1	6.2, 12.8
Respiratory 1600–1659	7	4.6	1.9, 9.5
Bone 1700–1709	60	41.1	31, 53.5
Soft tissue 1710–1719	17	16.1	9.4, 25.8
Breast (female) 1740–1749	14	3.1	1.7, 5.1
Brain/CNS 1910–1929	44	12.4	9, 16.6
Endocrine 1930–1949	14	13.4	7.3, 22.5
Leukaemia 2040–2089	23	5.8	3.6, 8.6

SMN=second malignant neoplasm; O=observed; SIR=standardised incidence ratio; CI=confidence intervals; CNS=central nervous system.

reports the SIR associated with specific types of SMN. The cumulative risk of developing an SMN within 20 years of 3-year survival from the first cancer was 3.1% (s.e.=0.2%) and within 25 years was 4.2% (s.e.=0.3%). The overall AER was 1.2 extra cancers per 1000 survivors per year.

As the markedly increased risk of SMN following heritable retinoblastoma is well established, this group was excluded, and will be the subject of a separate report. Furthermore, in view of uncertainty about the completeness of ascertainment of family pedigree information in cases recorded as nonheritable retinoblastoma, we have excluded all cases of retinoblastoma as FPT (total 1089) from subsequent analyses.

### Nonretinoblastoma childhood cancers

In all, 15 452 cases were included in this cohort, with 147 163 person-years of observation accrued, a mean follow-up of 9 years and 6 months and a median follow-up of 7 years and 2 months. Table 2

**Table 2:** Risk of developing an SMN and selected sites in 3-year survivors of nonretinoblastoma childhood cancer

	**Nonretinoblastoma cohort (*n*=15 452)**		
**SMN sites and ICD9 codes**	**O**	**SIR**	**95% CI for SIR**
All (exc skin) 1400–2089 (exc 1730–1739)	201	5.8	5.0, 6.7
Digestive 1400–1599	27	8.8	5.8, 12.9
Respiratory 1600–1659	6	4.8	1.7, 10.3
Bone 1700–1709	33	25.2	17.3, 35.4
Soft tissue 1710–1719	15	15.9	8.9, 26.2
Breast (female) 1740–1749	11	2.8	1.4, 5.0
Brain/CNS 1910–1929	42	13.4	9.6, 18.1
Endocrine 1930–1949	13	13.9	7.4, 23.8
Leukaemia 2040–2089	23	6.5	4.2, 9.8

SMN=second malignant neoplasm; O=observed; SIR=standardised incidence ratio; CI=confidence intervals; CNS=central nervous system.

gives the overall SIR for any SMN and the SIRs for specific sites of SMN. A total of 201 SMNs occurred during the study period (excluding nonmelanomatous skin cancers) against 34.7 expected giving a SIR of 5.8 (95% CI: 5.0, 6.7). Among specific sites of SMNs, the highest SIR was for bone cancer (SIR=25.2) followed by soft tissue (SIR=15.9), endocrine (SIR=13.9) and brain and CNS (SIR=13.4) sites. The cumulative risk of developing an SMN within 20 years of 3-year survival was 2.8% (s.e.=0.24%).

### By duration of follow-up

Variation in risk of SMN with time from diagnosis was examined in the following periods: 3–9, 10–19, 20–29 and 30 years or more survived from diagnosis. The number of survivors entering each risk interval, the observed and expected number of SMNs occurring and the SIR and AER for each of these follow-up groups are given in Table 3

**Table 3:** Risk of SMN after nonretinoblastoma childhood cancer, by duration of follow-up from original diagnosis

	**Follow-up period from diagnosis (in years)**			
	**3–9**	**10–19**	**20–29**	**30 or more**
Number entering risk period	15 452	7862	2806	808
Person-years accrued during risk period	76 594	50 685	16 695	3189
Observed number of SMNs	92	64	34	11
SIR (O/E)	10.2	5.7	3.5	2.4
95% CI for SIRs	8.3, 12.6	4.4, 7.3	2.4, 4.9	1.2, 4.3
AER per 1000 survivors per year	1.1	1.0	1.5	2.0

SMN=second malignant neoplasm; O=observed; E=expected; SIR=standardised incidence ratio; AER=additive excess risk.

Statistical test for trend in SIR (unadjusted), *P*<0.001.

Statistical test for trend in SIR (adjusted), *P*=0.002.

Statistical test for heterogeneity in SIR (unadjusted), *P*<0.001.

Statistical test for heterogeneity in SIR (adjusted), *P*=0.006.

.

Univariate analysis demonstrated a statistically significant decline (*P*<0.001) in the SIR over successive decades from diagnosis (Table 3), persisting after adjusting for possible confounding (*P*=0.002) in a multivariate model also including treatment, era of diagnosis and age at diagnosis of the first cancer. There was a statistically significant excess SIR within each decade of follow-up, the excess number of cancers remaining between one and two extra cases per 1000 survivors per year over all follow-up intervals.

SMNs at three sites demonstrated a statistically significant trend in SIR with duration of follow-up: brain and central nervous system, female breast and myeloid leukaemia (Table 4

**Table 4:** Observed numbers, SIRs and 95% CIs for selected SMNs after nonretinoblastoma childhood cancer by duration of follow-up from the first cancer diagnosis.

		**Sites of SMN**								
		**Leukaemia**	**Digestive**	**Bone**	**Soft tissue**	**Breast (female)**	**Genital organs (female)**	**CNS**	**Thyroid and endocrine**	**Myeloid leukaemia**
	ICD9 code	2040–2089	1400–1599	1700–1709	1710–1719	1740–1749	1790–1849	1910–1929	1930–1949	2050–2059
Duration of follow up from the first cancer diagnosis (in years)										
3–9	O	20	6	20	5	0	1	28	4	15
	SIR	10.0	13.4	27.4	13.0		3.2	18.5	14.8	28.9
	95% CI	6.1, 15.4	4.9, 29.2	16.7, 42.2	4.2, 30.3		0.1, 17.6	12.3, 26.9	4.0, 37.8	16.2, 47.8
10–19										
	O	2	9	11	8	6	2	9	8	1
	SIR	1.9	11.2	23.3	22.6	8.3	1.5	9.4	21.5	2.0
	95% CI	0.2, 7.0	11.6, 41.0	5.1, 21.3	9.7, 44.5	3.0, 18.0	0.2, 5.4	4.3, 17.9	9.3, 42.4	0.1, 11.3
										
20–29	O	1	7	2	1	4	5	5	0	1
	SIR	2.7	6.7	22.0	6.2	2.1	2.9	9.9		4.0
	95% CI	0.1, 15.2	2.7, 13.8	2.7, 79.6	0.2, 34.7	0.6, 5.3	1, 6.8	3.2, 23.1		0.1, 22.3
										
30 or more	O	0	5	0	1	1	1	0	1	0
	SIR		6.5		21.7	0.8	1.6		16.7	
	95% CI		2.1, 15.2		0.6, 121.0	0.02, 4.6	0.04, 9.0		0.4, 92.8	
										
	Test for trend	*P*=0.1	*P*=0.137	*P*=0.483	*P*=0.978	*P*=0.016	*P*=0.982	*P*=0.017	*P*=0.33	*P*=0.001

O=observed; SIR=standardised incidence ratio; CI=confidence intervals; CNS= central nervous system.

). The risk of myeloid leukaemia was greatest within the first decade from diagnosis and the later decline in risk was quite striking, with SIRs of 28.9, 2 and 4, respectively, over the first three decades of follow-up.

### By treatment of first cancer

Survivors were classified into four treatment categories: neither RT nor CT, RT only, CT only and both RT and CT. There was evidence of heterogeneity among the SIRs for any SMN (*P*<0.001), with the greatest excess risk in cases receiving both RT and CT (SIR 12.5, 95% CI: 9.8, 15.8). This evidence of significant heterogeneity persisted on multivariate analysis, which adjusted for the effect of the other factors on treatment (Table 5

**Table 5:** SIRs and observed numbers of developing a second malignant neoplasm following all nonretinoblastoma childhood cancers, and selected sites by treatment received, era of diagnosis and age at diagnosis of the first cancer

	**Sites of SMN**								
	**All SMNs**	**Leuk**	**GI**	**Bone**	**Soft tissue**	**Breast (female)**	**CNS**	**Endocrine**	**Myeloid leuk**
*Treatment received for primary cancer*									
Neither RT nor CT	3.3 (26)	1.5 (1)	6.9 (5)	12.8 (3)	0 (0)	0.9 (1)	11.4 (7)	4.7 (1)	3.6 (1)
RT only	6.0 (91)	3.6 (4)	10.4 (17)	31.8 (13)	17.6 (6)	4.2 (9)	13.6 (15)	22.0 (8)	5.8 (3)
CT only	7.4 (12)	11.9 (3)	21.1 (2)	32.0 (3)	16.9 (1)	0 (0)	10.0 (2)	0 (0)	26.1 (2)
Both RT and CT	12.5 (72)	14.6 (15)	9.9 (3)	34.4 (14)	34.5 (8)	6.7 (1)	22.2 (18)	22.2 (4)	38.0 (11)
Test for heterogeneity (unadjusted)	*P*<0.001	*P*<0.001	*P*=0.59	*P*=0.45	*P*=0.08	*P*=0.33	*P*=0.29	*P*=0.29	*P*<0.001
Test for heterogeneity (adjusted)	*P*=0.003	*	*	*	*	*	*	*	*
									
*Era of diagnosis*									
Pre-1970	3.6 (83)	2.0 (3)	6.9 (17)	17.2 (9)	14.8 (7)	2.2 (8)	8.2 (13)	11.0 (6)	2.7 (2)
1970–1979	9.8 (90)	6.5 (9)	12.1 (6)	35.7 (21)	20.1 (7)	10.1 (3)	21.8 (24)	20.2 (6)	11.4 (5)
1980–1987	10.6 (28)	18.5 (11)	35.2 (4)	14.9 (3)	8.1 (1)	0 (0)	10.9 (5)	10.9 (1)	69.9 (10)
Test for trend (unadjusted)	*P*<0.001	*P*<0.001	*P*=0.004	*P*=0.61	*P*=0.875	*P*=0.032	*P*=0.132	*P*=0.590	*P*<0.001
Test for heterogeneity (unadjusted)	*P*<0.001	*P*<0.001	*P*=0.005	*P*=0.093	*P*=0.639	*P*=0.045	*P*=0.01	*P*=0.541	*P*<0.001
Test for trend (adjusted)	*P*=0.093	*	*	*	*	*	*	*	*
Test for heterogeneity (adjusted)	*P*=0.10	*	*	*	*	*	*	*	*
									
*Age at diagnosis*									
0–4	7.9 (71)	5.7 (9)	16.3 (9)	26.2 (13)	16.0 (5)	4.2 (2)	17.4 (22)	23.0 (6)	14.1 (6)
5–9	5.8 (54)	3.2 (3)	6.6 (5)	23.6 (10)	14.8 (4)	0 (0)	15.1 (13)	7.8 (2)	5.3 (2)
10–14	4.6 (76)	11.2 (11)	7.4 (13)	25.7 (10)	16.5 (6)	3.6 (9)	6.9 (7)	12.1 (5)	16.9 (9)
Test for trend (unadjusted)	*P*=0.001	*P*=0.14	*P*=0.102	*P*=0.948	*P*=0.954	*P*=0.589	*P*=0.033	*P*=0.296	*P*=0.632
Test for heterogeneity (unadjusted)	*P*=0.005	*P*=0.08	*P*=0.117	*P*=0.967	*P*=0.985	*P*=0.174	*P*=0.084	*P*=0.310	*P*=0.294
Test for trend (adjusted)	*P*=0.031	*	*	*	*	*	*	*	*
Test for heterogeneity (adjusted)	*P*=0.09	*	*	*	*	*	*	*	*

GI=digestive; CNS=central nervous system; Leuk=leukaemia.

* Indicates where numbers were insufficient for multivariate analysis.

). Among SIRs for SMNs at selected sites, there was significant heterogeneity for secondary leukaemia (*P*<0.001), in particular myeloid leukaemia (*P*<0.001) (Table 5).

The SIR associated with treatment was initially calculated only for the 82% of nonretinoblastoma cases in which treatment information was available. However, since the treatment records of patients with SMN were sought with much more effort than for those not developing an SMN, such an analysis may be biased. The SIR analysis was therefore repeated including the ‘no record’ cases and assuming that all those for whom treatment details were not available received the treatment being analysed, but the results were not substantially different to those reported above (data not shown).

### By era and age at first cancer diagnosis

The risk of SMN was calculated for three main periods of diagnosis of the first cancer: pre-1970, 1970–1979 and 1980 and later. The SIR for all SMNs and specifically of the digestive tract and female breast demonstrated a significant trend of increasing excess risk with the more recent era of diagnosis (Table 5), particularly for secondary myeloid leukaemia: (*P*<0.001) across the diagnostic periods pre-1970, 1970–1979 and 1980 onwards, for which the SIRs were 2.7, 11.4 and 69.9, respectively. However, when the SIR for all SMNs was adjusted for other risk factors, the significant trend seen on univariate analysis did not reach formal statistical significance (*P*=0.093).

Table 5 shows risk of SMN by age at diagnosis of the first cancer. The SIR for SMN overall was greater with the first cancer under the age of 5 years, a significant trend being observed in the SIR on univariate analysis, which remained after adjustment for the effect of the other factors detailed in the Statistical methods section (*P*=0.031).

### Risk of SMN after selected types of first cancer

The SIRs of developing an SMN and the cumulative risks of developing an SMN within 20 years of 3-year survivorship of specific first cancer diagnoses are displayed in Table 6

**Table 6:** Risks of developing an SMN after selected types of first cancer

	**First cancer diagnosis**						
	**Brain and CNS tumours**	**Acute lymphoblastic leukaemia**	**Wilm's tumour**	**Hodgkin's disease**	**Soft tissue tumours**	**Neuroblastoma**	**Bone tumours**
Number in group	4009	3988	1298	1294	1086	660	638
Person-years accrued during study period	43 970	24 620	15 621	12 425	12 445	7827	5805
Mean follow-up (years and months)	10y 11m	6y 2m	12y	9y 7m	11y 5m	11y 10m	9y 1m
Observed number of SMNs	55	17	19	32	14	7	16
SIR (O/E)	4.7	5.3	6.9	9.2	4.3	5.0	8.4
95%CI for SIR	3.6, 6.2	3.1, 8.4	4.2, 10.8	6.3, 13.0	2.3, 7.2	2.0, 10.4	4.8, 13.6
AER per 1000 survivors	1.0	0.6	1.0	2.3	0.86	0.72	2.4
Cumulative risk of SMN within 20 years of 3-year survival	2.6%(s.e.=0.4%)		2.8%(s.e.=0.8%)		2.2%(s.e.=0.6%)	1.7%(s.e.=0.8%)	

SMN=second malignant neoplasm; O=observed; SIR=standardised incidence ratio; CI=confidence intervals; AER=additive excess risk; CNS=central nervous system; s.e.=standard error.

. The cumulative risks are provided for only those specific types of first cancer for which the mean period of follow-up exceeded 10 years.

## DISCUSSION

### After all childhood cancers combined

Our analysis of the long-term risks of SMN in a large, population-based national cohort of 3-year survivors of childhood cancer diagnosed between 1926 and 1987 develops previous work covering part of this cohort followed-up until the end of 1981 (Hawkins *et al*, 1987); an additional 8 years of such survivors (over 6000) have now been added, more than doubling the total period of person-years of follow-up. The mean period of follow-up of 10 years is substantially greater than other previously studied population-based cohorts. Large population-based Nordic studies of SMNs covered mean durations of follow-up of 6 years (Olsen *et al*, 1993) and 7.5 years (Garwicz *et al*, 2000). Continued follow-up provides an opportunity to assess long-term trends in risk and helps clarify uncertainty, which still exists for long-term survivors.

In our overall cohort (including retinoblastomas), 278 SMNs were observed in total with 245 excluding melanomatous skin SMNs, which was approximately six-fold the number expected. The cumulative risk of developing an SMN within 25 years of 3-year survival was 4.2%. This result is comparable with the previous report on the UK cohort as well as other population-based series. In the former, a six-fold increased risk was found of SMNs and a cumulative risk by 25 years from the 3-year survival of 3.7% (Hawkins *et al*, 1987). A population-based cohort study of 30 880 childhood cancer survivors from the Nordic cancer registries found 247 SMNs, a SIR of 3.6 and a cumulative risk of SMN by 25 years from diagnosis of 3.5% (Olsen *et al*, 1993). Similar results were described in Italy (Magnani *et al*, 1996). It has previously been noted that early hospital-based series yielded substantially higher risks of SMN than population-based series (Hawkins and Stevens, 1996). Early hospital-based studies of SMNs after childhood cancer, carried out by the Late Effects Study Group (LESG) reported a SIR of SMN of 15 and a cumulative risk at 20 years from diagnosis of 12% (Mike *et al*, 1982; Meadows *et al*, 1985). However, the Childhood Cancer Survivor Study, one of the largest and most comprehensive of treatment-centre-based cohort studies of SMNs, in the follow-up of 13 581 survivors of childhood cancer for a median of 15 years from the diagnosis of the first cancer, a SIR of 6.38 and a cumulative risk of SMN by 20 years of 3.2% were reported (Neglia *et al*, 2001). There is consistency of risk estimates from the large European population-based series and more recent hospital-based studies.

The differences in risk estimates between large population-based and large early hospital-based series may reflect different therapeutic practices and/or the potential for bias associated with hospital-based studies, for healthy survivors are more likely to become lost to follow-up than unhealthy survivors who need to return for further care, as discussed in detail in relation to large hospital-based studies of SMNs after childhood Hodgkin's disease (Wolden *et al*, 1998).

### After nonretinoblastoma childhood cancers

One of the strengths of this study is the length of follow-up, thus providing accurate estimates of risks of SMN in successive decades from diagnosis. We have demonstrated a significant decline in SIR of SMN with increasing duration of follow-up, which was not evident in our previous analysis (Hawkins *et al*, 1987), highest (10.2) 3 to 9 years after diagnosis with a significant decline in SIR over subsequent decades. A similar decline in SIR has been recently reported in another study of survivors of childhood cancer (de Vathaire *et al*, 1999), and also in a comprehensive study of populations irradiated in childhood, including the survivors of the atomic bombs in Japan (Little *et al*, 1991). However, perhaps of particular note for clinicians and the survivors themselves is the AER by duration of follow-up. The LESG reported a marked rise in the AER of SMN with duration of follow-up: 1.5 extra cancers per 1000 survivors per year within 2 to 4 years of the first cancer, which increased to 15 extra cancers after 20 years (Tucker *et al*, 1984). Although direct comparison is not possible, in our study, within the first decade from diagnosis of the first cancer, 1.1 extra cancers were observed per 1000 survivors per year increasing to only 1.5 extra cancers per 1000 survivors per year beyond 20 years from diagnosis.

The risk of SMN was influenced by the era of diagnosis of the first cancer, and our categories (pre-1970, 1970–1979 and 1980 onwards) crudely reflect the change in treatment practice within British paediatric oncology. Prior to 1970, there was little chemotherapy used, in the 1970s single-agent chemotherapy and early treatment protocols were developed and since 1980 there has been a more widespread use of multiagent chemotherapy. The SIR rose from 3.6 following treatment in the pre-1970 era to 10.6 among those treated from 1980 onwards, a trend to which digestive system and breast cancers contributed. Numbers of SMNs in these groups were small, but there was a highly significant trend (*P*<0.001) in the risk of secondary myeloid leukaemia with a striking increase in the SIR from 2.7 in those treated pre-1970 to 69.9 in the group treated after 1979. All these second primary myeloid leukaemias were acute, such that the SIRs reported underestimate the excess risk of such leukaemias. The Nordic cohort study reported a similar significant trend with a calendar period of diagnosis for all SMNs, with a trend also in the second leukaemia group (Olsen *et al*, 1993). In the present data, the association between SMN risk and era of diagnosis of the first cancer was not formally statistically significant in a multivariate model that included type of treatment for first cancer, suggesting that this association was due to the confounding effect of the recent intensive treatment regimes. Acute myeloid leukaemia, the most frequent of the therapy-related leukaemias, has been associated with treatment of the first cancer with exposure to alkylating agents, epipodophyllotoxins and irradiation (Hawkins *et al*, 1992; Sandoval *et al*, 1993). We found substantial heterogeneity in SIR for SMN in relation to exposure to neither, either radiotherapy or chemotherapy or both, with the greatest risk observed in probably the most intensive treatment category (both chemotherapy and radiotherapy).

Since 1987, treatment protocols have intensified with treatment given to the limits of patients' toleration. It is therefore of great concern that the trend of increasing excess risks of SMN apparent in our data may be even greater in subsequent decades of survivors. It is important therefore that the outcome data of childhood cancer continue to be monitored in order to identify subgroups at particular risk of SMN. It is important to note, however, that in comparison with almost certain death from untreated childhood, the absolute risks of SMN experienced by survivors are small.
